# Numerical and experimental analysis of thermal behaviour of high voltage power cable in unfilled ducts

**DOI:** 10.1038/s41598-024-71281-x

**Published:** 2024-09-04

**Authors:** Michele Quercio, Juan Carlos Del Pino Lopez, Sergio Grasso, Aldo Canova

**Affiliations:** 1 Roma Tre University, 00146 Rome, Italy; 2 University of Sevilla, 41013 Seville, Spain; 3 Be Shielding S.r.l., 10098 Cascine Vica Rivoli, Italy; 4 Polytechnic University of Turin, 10129 Turin, Italy

**Keywords:** Electrical and electronic engineering, Power distribution

## Abstract

The work addresses the topic of the thermal study of high-voltage power cables installed inside plastic pipes in the absence of filling. The presence of air inside the pipe creates an insulating layer that does not favor heat exchange and makes the calculation of the flow rate more complex, as it is necessary to take into account the thermal phenomena of natural convection and radiation between the surface of the cable and the internal surface of the tube. The numerical model based on the finite element calculation was compared with the experimental results obtained on a simulacrum in which the temperatures on the different layers of the cable were measured. After this validation, some typical installation configurations of single and double energy transport triads were analyzed.

## Introduction

The thermal analysis of electrical cables, with particular reference to high voltage ones, represents an essential phase in the design of an electrical line to evaluate the power line’s ampacity. Many factors influence the thermal behavior and, therefore, the ampacity, mainly the physical characteristics of the materials that make up the cable and the type of laying. Based on this information, the equations governing heat transfer may or may not include convection and radiation phenomena in addition to the conductive heat exchange, which is always present. Solution equations are differential equations that can be solved using various analytical or numerical methodologies or a mix of both^1^. In the case of cables laid directly in the ground, the problem is relatively simple and governed by conductive heat exchange equations with a possible convective exchange on the ground’s surface. In this case, the analytical methods are reliable and have been used for many years for power line design. The standard IEC 60287^2^ establishes the methods of calculating the rated current of the cables in a range of different installation conditions. The analytical equations given in the standard are based on the fundamental theory of heat transfer, while others are empirical or semi-empirical equations derived from “field” experience. The standard includes an empirical approach to determining rated currents for cables in unfilled channels. In many situations, the IEC approach does not consider some environmental parameters or their variation. To improve the ampacity calculation’s reliability or the temperature distribution estimation, the numerical integration of the heat transfer equations must be followed. The use of numerical approaches like the Finite Element Method has been adopted since 20 years ago^3–5^, in many cable configurations. Different comparisons have been provided in standard layouts, where the cables are in contact with soil materials. In^6^, where a comparison between the analytical approach, based on IEC formulae, and FEM, is presented, the numerical solution produces more reliable results which better take into account the wind contribution. Analyzing the literature on the paper topic, we observe many works related to heat transfer performance for underground cables, mainly for directly underground cables. On the contrary, studies on cables installed in unfilled pipes are relatively few. The aspects related to heat transfer by convection and radiation have yet to be sufficiently studied from a theoretical and experimental point of view. In the case of cables positioned in free air or inside confined spaces in the air, e.g., pipes or tunnels, convective and radiation heat exchange phenomena also occur, and the complexity of the domains and the equations increases considerably. In this case, analytical methods are challenging to apply, and in the past, semi-empirical methods based on experimental tests have been used. Today, the possibility of exploiting numerical computational codes that implement the described physics allows us to solve these thermal problems more accurately, avoiding an empirical approach. The installation of cable in free air is considered weekly by the standards, which take into account effects like the speed of wind in the direction only selectively. To consider such effects, together with solar radiation, the paper^7^ presents the impact of selected weather conditions on the current-carrying capacity of power cables. The calculation is based on computational fluid dynamics (CFD) simulations performed using ANSYS Fluent software. A similar approach and the same software, on a more manageable configuration of only one conductor (aerial and without insulation) but in a 3D geometry domain, has been adopted in the paper^8^. In paper^9^, the study of a group of power cables in free air is faced with a numerical approach (COMSOL Multiphysics^10^). The paper proposes a thermal-electrical circuit model for the accurate steady-state rating for the most common installation configurations, and the results are compared with IEC, FEM, and experimental tests. The complexity of the numerical problem, also in terms of time calculation and convergence, increases in the case of cables installed in confined spaces, and in particular, this deals with installing cables inside unfilled plastic conduits. Neher and McGrath made the first evaluations of this type of installation^11^ in 1957 and later taken up in the IEC 60287 standard. The paper^12^ the numerical analysis of heat dissipation from a set of cables installed on the floor of a shallow trough is analysed. The cover of the concrete trough is exposed to the ambient air and the sun. The floor and the walls, also made of concrete, are in contact with compacted rock and native soil. The paper compares the FEM approach with the IEC standard 60287 and an analytical approach, like Slaninka’s method^13^. Recently some papers have presented a numerical approach based on FEM to the study of power cables inside unfilled ducts. First papers^14–16^ deal with the case of a three-phase power line installed inside an unfilled pipe. The work is theoretical and shows the influence of some parameters, such as the effect of domain mesh on the distribution of air velocities and temperature and the effect of the vertical position of the cables inside the tube. The papers^17–21^ show a significant discrepancy between the IEC standard calculations for steady-state conditions. The comparison was also carried out with experimental tests and FEM simulations. The discrepancies between the experimental evaluations and the regulatory approach are mainly attributable to the calculation of the heat transfer of the air between the surface of the cable and the internal surface of the duct. Starting from the presented state of art^22–35^ in this paper, a 2D numerical analysis of three-phase power lines with each cable placed inside a pipe is analyses^36–38^. In order to provide proper validation, an experimental setup has been realized, and the model simulation results are compared with the experimental test. In particular, attention has been dedicated to simulating the thermal dissipation linked to convective and radiation contribution. After the validation stage, a numerical parameter analysis of some power line configurations is presented and discussed. The work is structured as follows: “Experimental results of the power cable” section, a description of the power cable model, is introduced, and the experimental setup is described. In “Numerical model” section, the numerical model simulation is discussed. “Comparison between numerical and experimental results” section presents the comparison between numerical and experimental results. Following, “Three-phase configuration” section introduces the three-phase configuration. Finally, conclusions are drawn in “Conclusion” section.

## Experimental results of the power cable

### Description of the power cable model

**Fig. 1:**
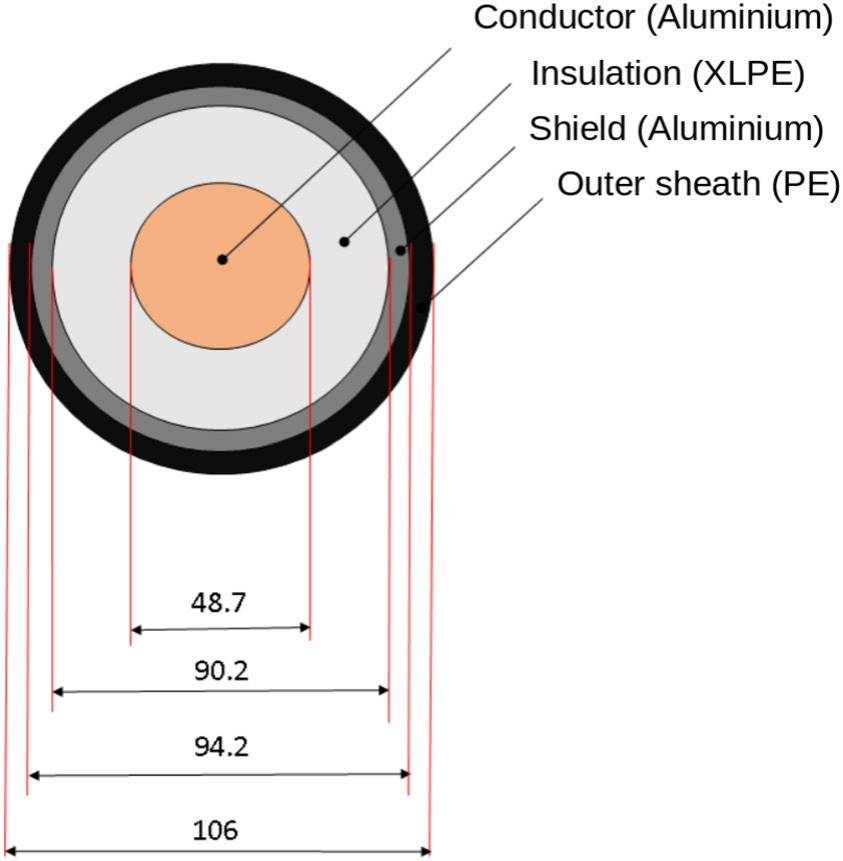
Power cable dimensions.

**Fig. 2:**
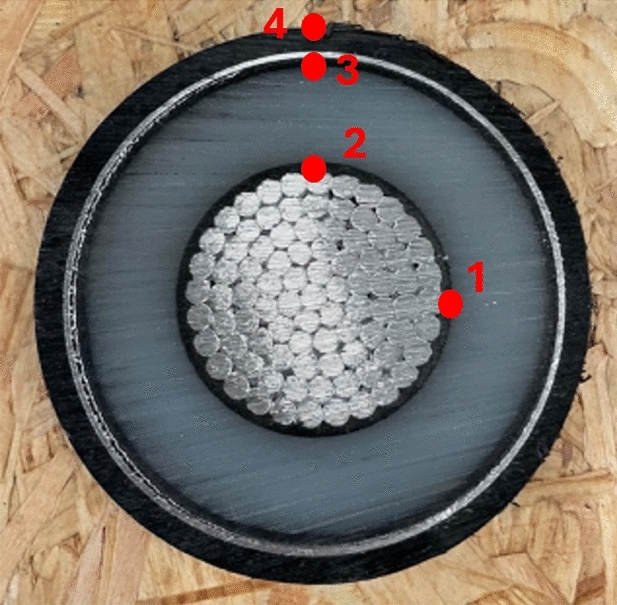
Cross-section cable.

The structure of the cable is shown in Figs. 1 and 2, which contains the Aluminium conductor, a layer of XLPE insulation, a layer of aluminum with the function of a shield, and an external PE insulation layer. The picture reports the main dimensions in millimeters. It is a 1600 mm^2^ aluminum cable, and under standard installation conditions, it has an ampacity of about 900–1000 A. The thermal properties of the materials for the cable simulations are those reported in the IEC standards 60853-2.

### Experimental set-up

**Fig. 3:**
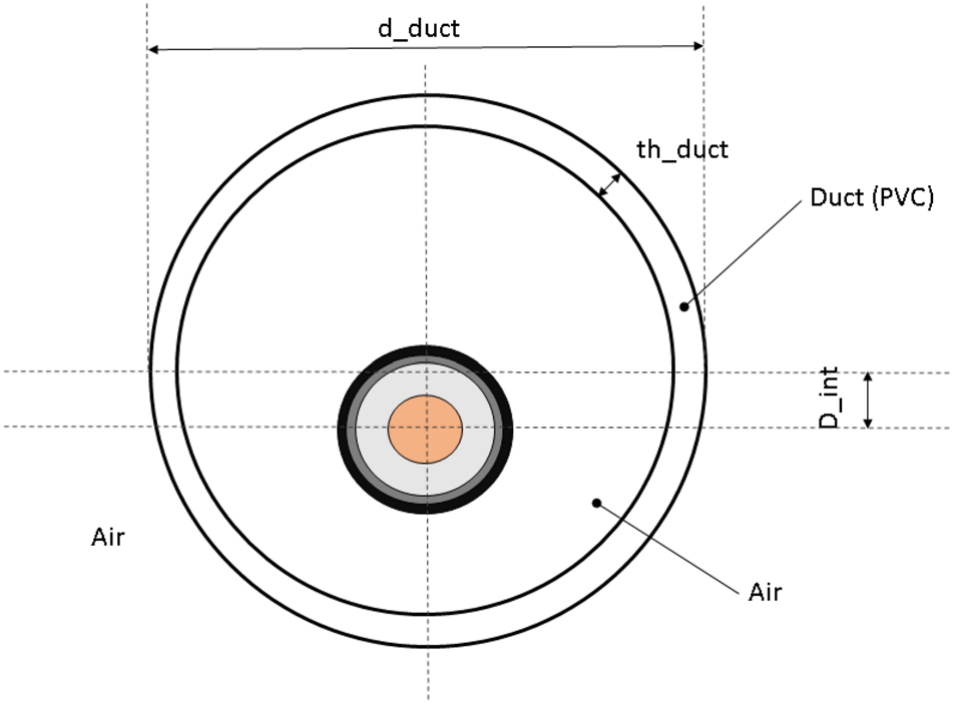
Set-up measurement.

In Fig. 3 is reported the scheme of the measurement set up where the power cable is inserted inside the duct and is centered along the vertical axis. The vertical position is a parameter $$(D_{int})$$ and two positions are chosen in the analysis: $$D_{int}=0 $$ and $$D_{int}=\frac{(d_{duct}-d_{cable})}{2}$$.

**Fig. 4:**
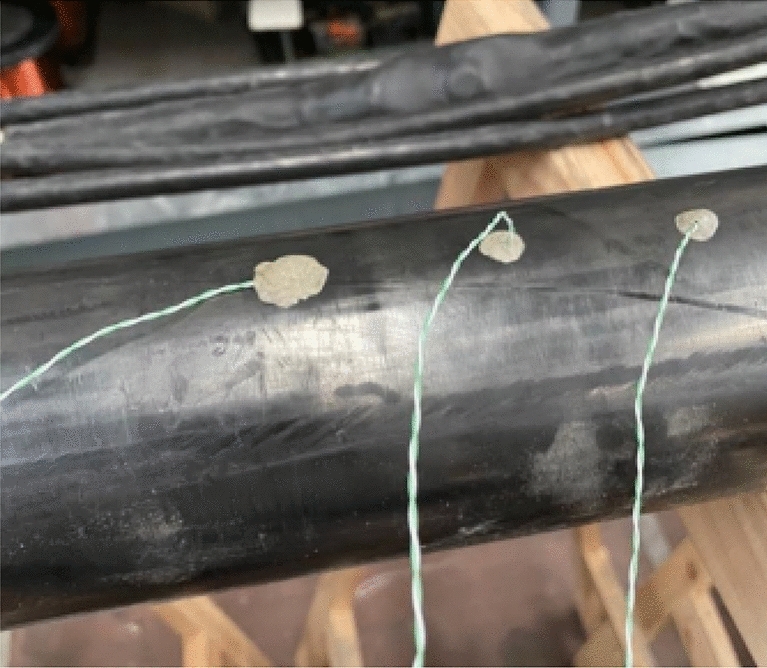
Probe positions in the pipe.

**Fig. 5:**
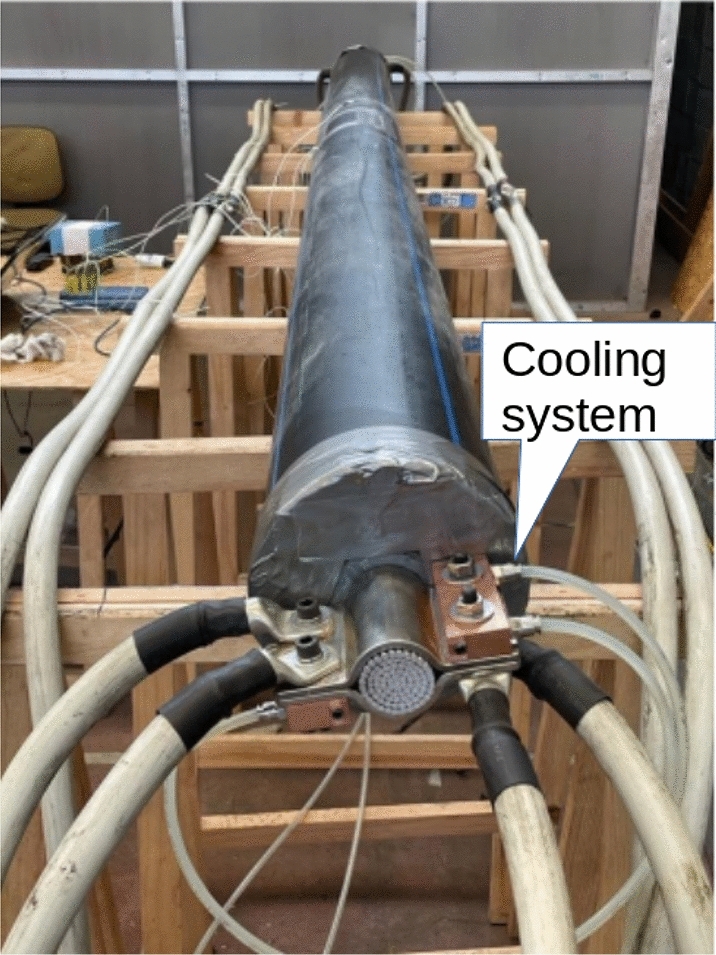
Cable centered inside the thermally insulated pipe.

The other parameter are: $d_{duct}=200$mm and $th_{duct}=5$mm. The total length of the analyzed cable is 4 meters. In order to evaluate the trend over time of the temperature of the various layers of the cable and of the pipe, probes were applied, as shown in Fig. 2. Figures 4, 5 show the set-up used for laboratory tests.

**Fig. 6:**
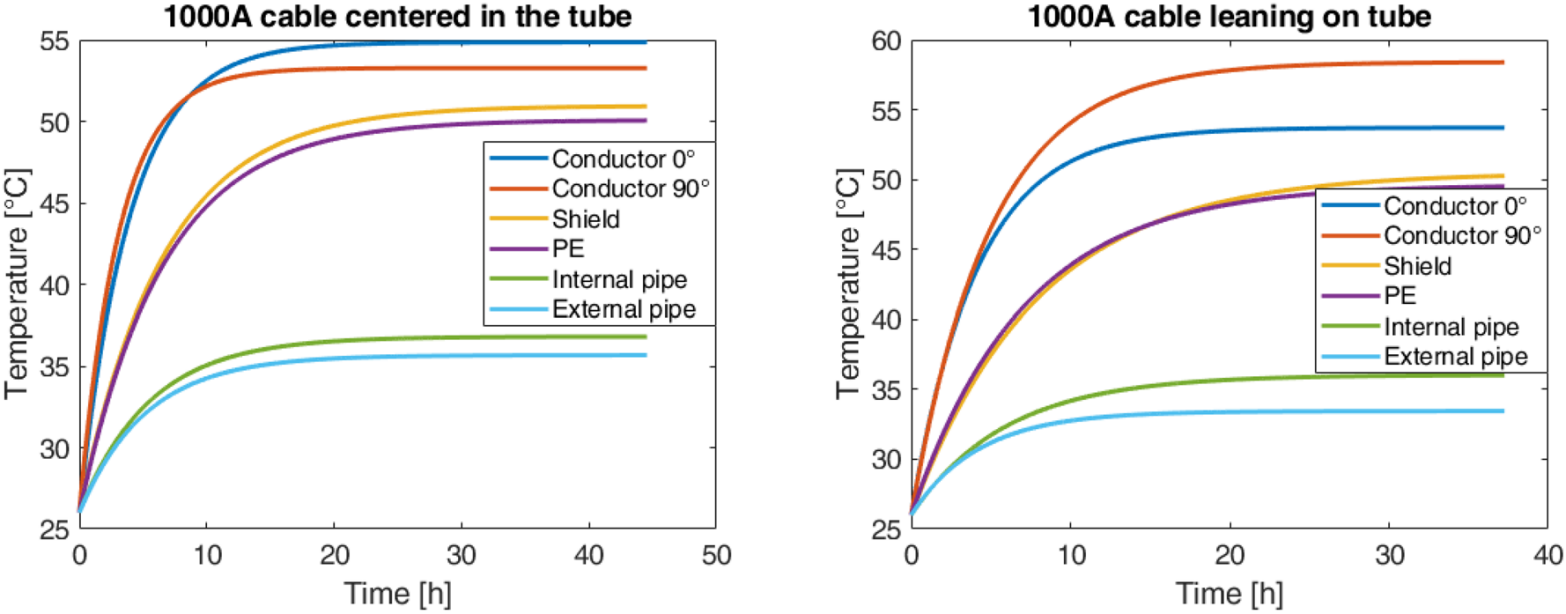
Overtemperature with 1000A.

**Fig. 7:**
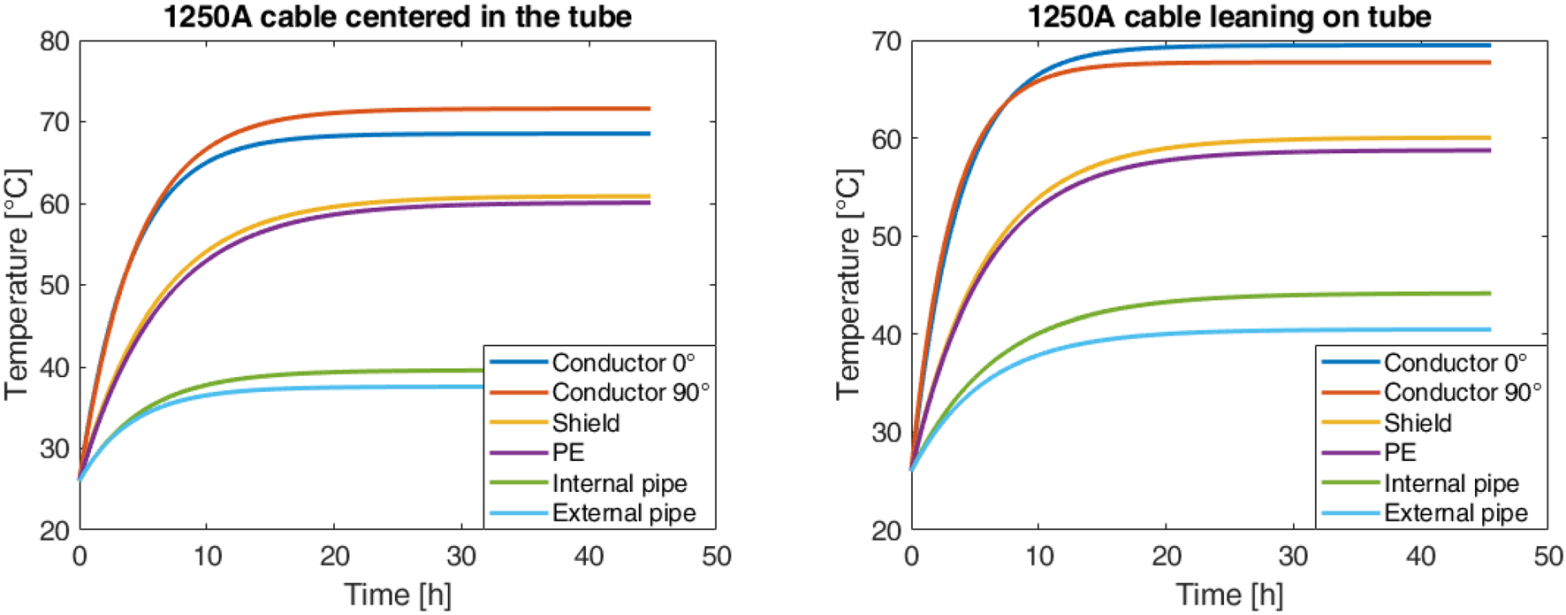
Overtemperature with 1250A.

The tests were carried out for two different current values, 1000A, and 1250A, each considered as configuration first the cable in the center of the tube and then the cable resting on the tube. The results are shown in Figs. 6 and 7.

In the tests, when powering the HV cable, the temperature increased at the inlet and outlet points of the current, which propagated towards the center of the cable, falsifying the values acquired by the thermocouples (cable small size). For this reason, a continuous cooling system has been positioned in the cable’s two ends, as seen in Fig. 5. By circulating water through the cable, heat generated at the ends of the cable can be dissipated more efficiently, resulting in a more uniform temperature distribution along the length of the cable, as shown in Table 1.

**Table 1:** Temperature evaluation along the cable in the case of 1250 A cable centered in the tube Conductor 90°.

Position	T [°C] w/o cooling system	T [°C] w/ cooling system
Left end	120	100
Center	100	100
Right end	120	100

Water cooling systems have been successfully implemented in various high-voltage power cable applications, including submarine and underground cables. These systems typically consist of a closed water loop circulated through the cable using pumps and heat exchangers. The water absorbs heat from the cable and then cools by passing through a heat exchanger before recirculating. Water cooling systems can provide several benefits for high-voltage power cables. First, they can eliminate the end effects that can occur when heat accumulates at the ends of the cable, leading to temperature gradients and potential damage to the insulation. Second, they can improve the overall thermal performance of the cable, allowing it to carry more current without overheating. Finally, they can increase the cable’s lifespan by reducing thermal stress and minimizing the risk of insulation failure.

## Numerical model

In order to analyze and examine the thermal behavior of the cable under different features and characteristics, a numerical model is designed in the 2D model using the commercial software Comsol^10^. The problem of the cable inside the tube was addressed through two studies: stationary and time-dependent. For each study, a multiphysics problem was solved by considering the physics of Heat Transfer in Solids and Fluids, Turbulent flow, Surface-to-surface radiation, and Laminar flow. For each of them, the boundary conditions have been defined:

1. *Heat transfer in solids and fluids:* A constant temperature equal to the ambient temperature was set to the external perimeter of the circle representing the external ambient.Furthermore, the conductor of the cable was considered as a heat source in wich the $Q_{0}$ is equal to:
  1 $$\begin{aligned} Q_{0}=\frac{W_{cable}}{S_{cable}} \end{aligned}$$
  Where
  - $$W_{cable}=R_{cable} \cdot I_{cable}^{2}$$
  - $S_{cable}$ is the cross-section of the cable
2. *Turbulent flow:* This physic was set to the air domain outside the pipe. A Wall boundary condition was selected considering the perimeter of the pipe and that of the external air domain. To consider the pressure constraint a point inside the air region was selected imposing 0 Pa on it.
3. *Surface-to-surface radiation:* Due to the high temperatures, the radiation between cable and pipe and between pipe and the external air domain is also considered.
4. *Laminar flow:* Finally, due to the geometry in the air region between the cable and the pipe, a laminar flow pattern is imposed.

After setting the problem both from a geometric and physical point of view, it was possible to carry out the analyzes first in a steady state and then in the transitory case. The regime of the latter must correspond to the results of the stationary case.

### Stationary study

**Fig. 8:**
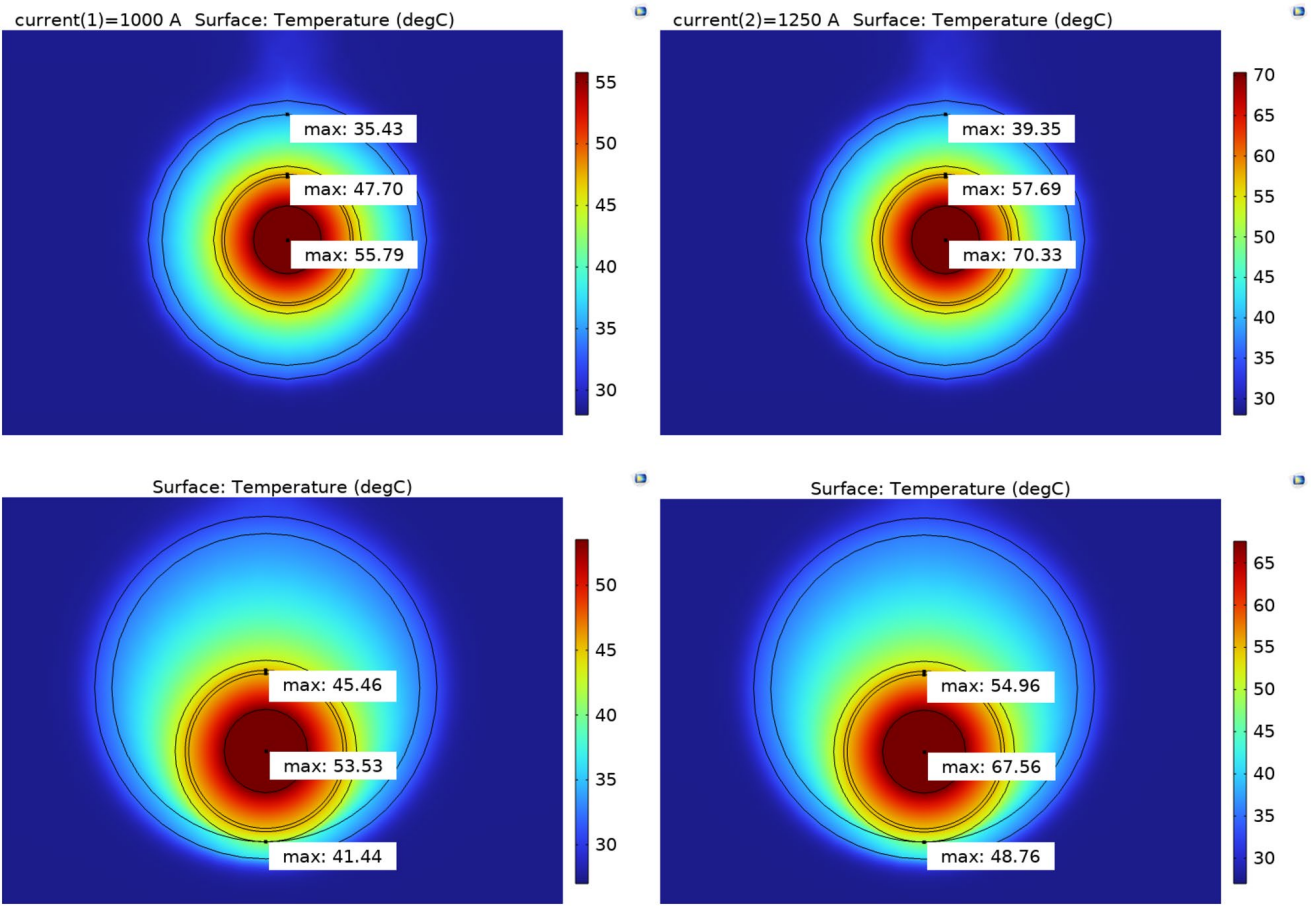
Temperatures of the cable respectively with currents of 1000 A and 1250 A centered in the tube and the same with the cable resting in the lower part of the tube (From top to bottom).

This subsection shows the simulations of the experimental case in the stationary condition. Figure 8 shows the operating temperatures of the case at 1000A and at 1250A in the condition of cable in the center of the pipe and resting on it.

### Time dependent study

**Fig. 9:**
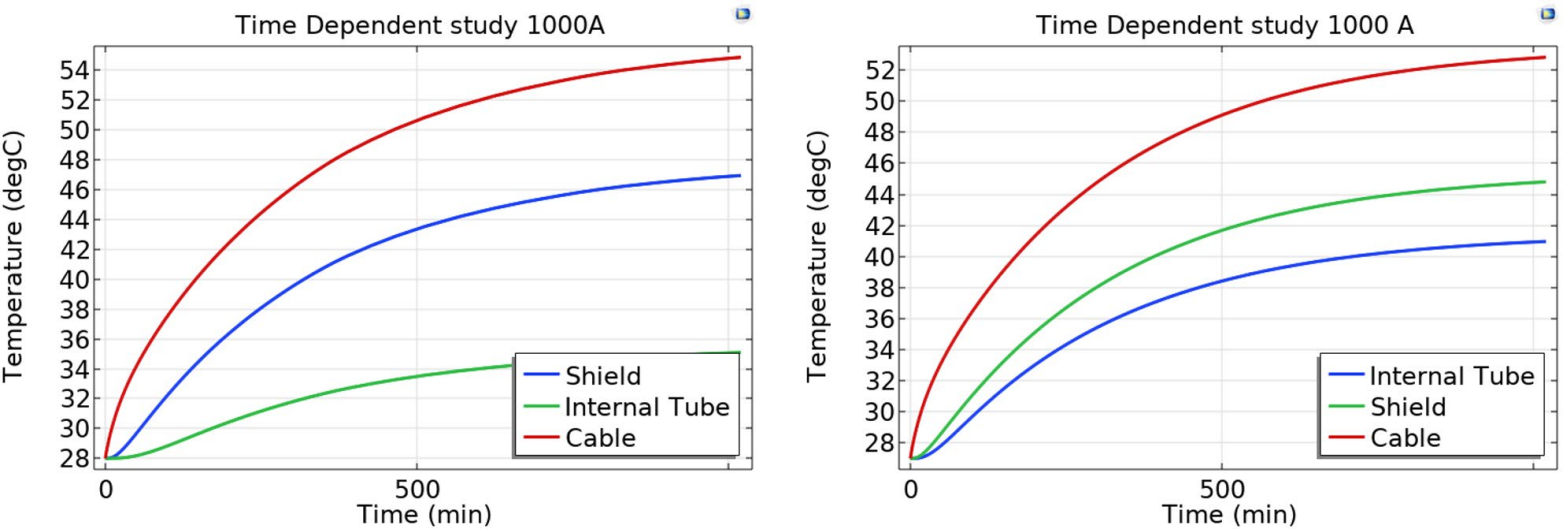
Temperatures Time Dependent Study 1000A cable centered in the tube and cable leaning against the tube.

The study of the temperature trend as a function of time is shown in Fig. 9. In order not to burden the reading, the values are reported considering only a current of 1000A. For reasons related to the calculation time, a value of 18 h was considered in which the steady state value begins to be reached.

From the graphs shown in the Figs. 8 and 9 it is noted how the steady state values of the transient temperatures coincide with the values of the stationary study. This allows to validate the goodness of the model.

## Comparison between numerical and experimental results

**Fig. 10:**
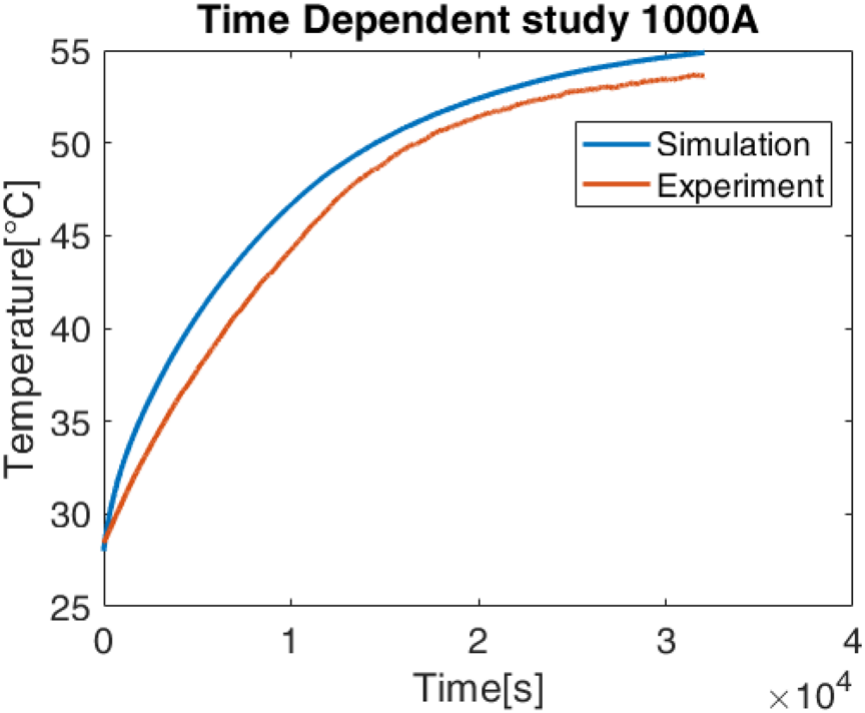
Comparison simulated and experimental data, cable centered in the tube.

**Fig. 11:**
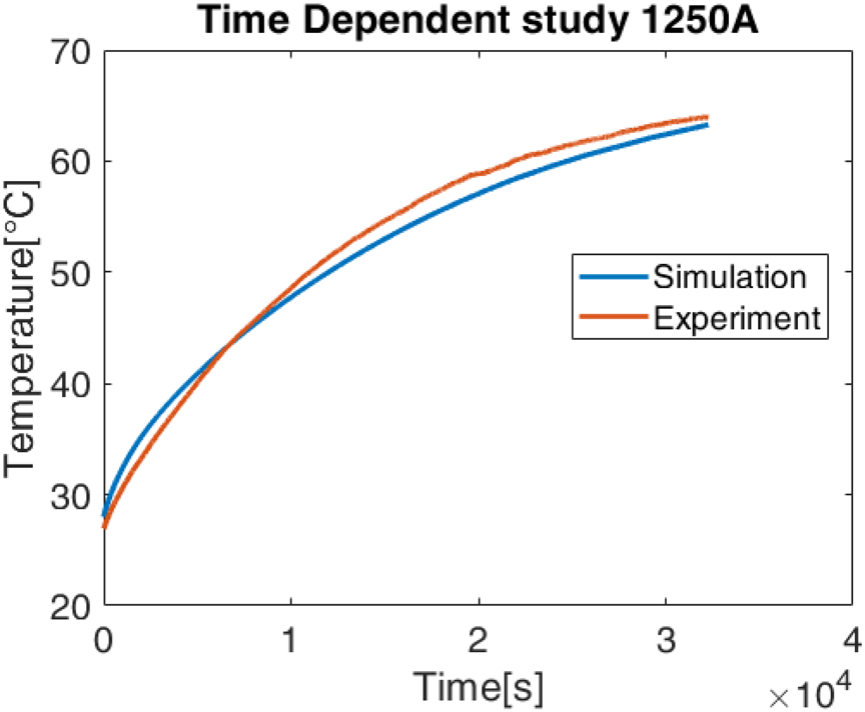
Comparison simulated and experimental data, cable centered in the tube.

Figures 10 and 11 show the temperature trends for the cable only in the simulated and experimental case in the cable condition at the center of the pipe for 1000 A and 1250 A. The comparison of the curves shows that the trends differ slightly from the experimental case so that the simulation fully reflects reality. In Tables 2 and 3 are reported to clarify the comparison of the temperature values of each layer of the cable and pipe for the different cases in the steady state condition.

**Table 2:** Comparison of the temperature between simulated and experimental case (1000 A).

Case	1000 A centered Exp., °C	1000 A resting Exp., °C	1000 A centered Sim., °C	1000 A resting Sim., °C
Conductor	56	54	55.8	53.55
Shield	47.77	44.3	47.7	45.5
Insulation	47	44.3	45.3	43
Pipe outer wall	36.1	33	33.3	31.2
Pipe internal wall	35.8	34.2	35.4	32.8

**Table 3:** Comparison of the temperature between simulated and experimental case (1250 A).

Case	1250 A centered Exp., , °C	1250 A resting Exp., , °C	1250 A centered Sim., , °C	1250 A resting Sim., , °C
Conductor	70	70.4	70.3	67.6
Shield	57.3	56.8	57.7	55
Insulation	56.3	55.7	53.4	52
Pipe outer wall	37.6	38.6	36	37
Pipe internal wall	40	41	40	38

## Three-phase configuration

**Fig. 12:**
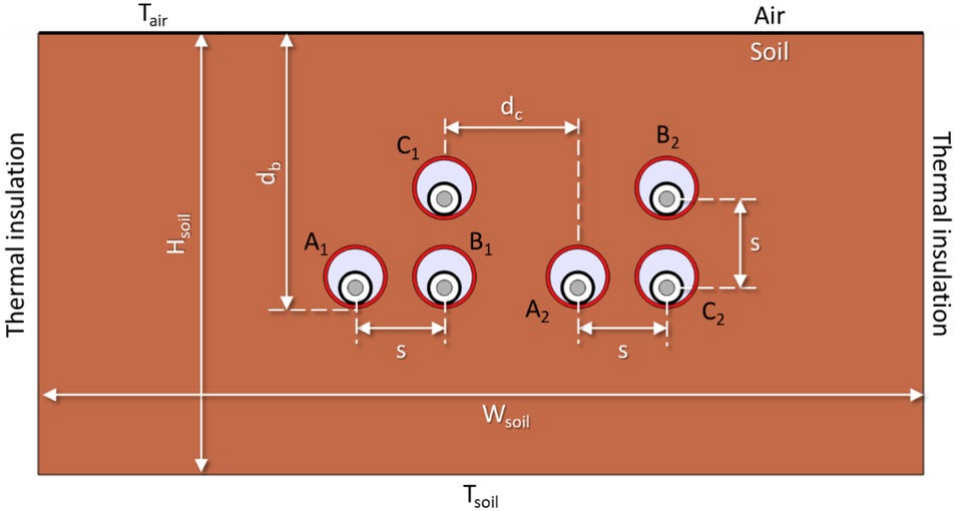
Main dimensions (parameters), phase arrangement and boundary conditions for the case study.

Once the numerical model of the cable inside the duct is validated, it is now possible to develop an in-depth parametric analysis on a typical two-circuit underground arrangement. In particular, the system shown in Fig. 12 is the reference case, where the same 1600 mm^2^ aluminum conductor employed in previous sections is considered. The values of the main parameters of the system are summarized in Table 4, where dp is the burial depth, s the clearance between phases, dc is the clearance between circuits, and Hsoil and Wsoil is the height and width of the soil domain, respectively. Phases arrangement for this case study is also described in Fig. 12, as well as the different boundary conditions applied for the thermal problem, being Tair, the temperature at the air-soil interface, and Tsoil, the soil temperature at a certain depth. In this situation, and assuming shields in single-point configuration, the ampacity of this case study is 883 A (phase current).

In the following, a parametric analysis is developed by varying each parameter in a reasonable range while the rest remain as shown in Table 4.

**Table 4:** Values for the parameters of Fig. 12.

Parameter	Value	Parameter	Value
$d_{b}$	1.6 m	$T_{soil}$	10 °C
S	0.28 m	$W_{soil}$	40 m
$d_{c}$	0.42 m	$H_{soil}$	20 m
$T_{air}$	20 °C	$k_{soil}$	0.833 $\frac{W}{m K}$

### Model assumptions

There are three main aspects to be considered during the modelling stage: the mesh size, the size of the soil domain and the type of boundary conditions to be applied.

#### Mesh size

**Fig. 13:**
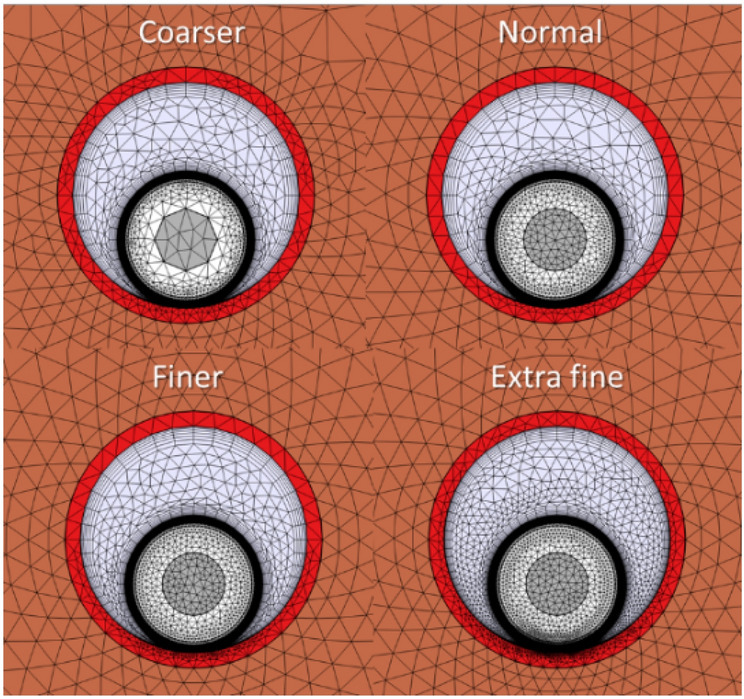
Mesh sizes considered in the study.

**Fig. 14:**
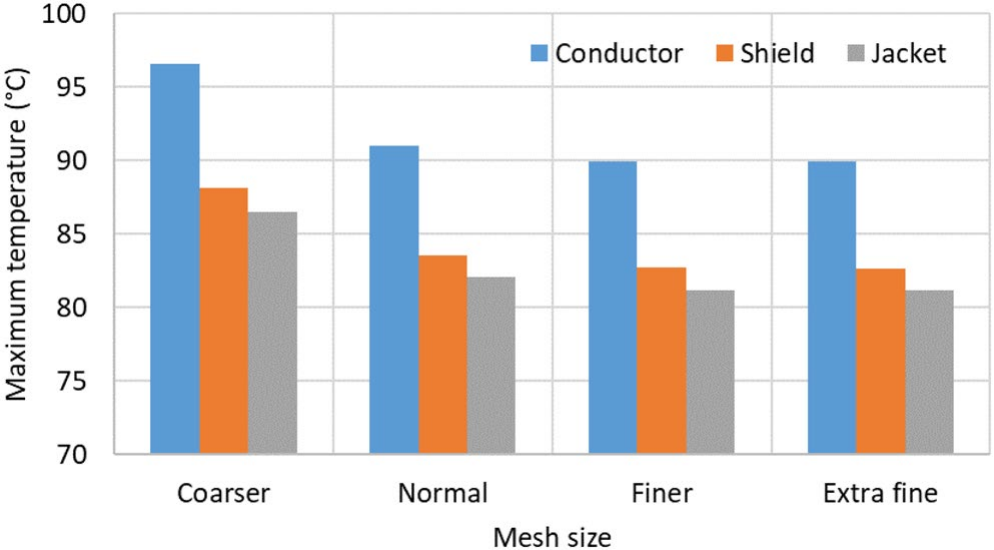
Temperature in conductor, shield and jacket depending on the mesh size (phase current of 883 A).

The mesh size is a crucial point to analyze before starting the parametric analysis since it influences both the computational cost and the accuracy of the results. In this sense, four different mesh sizes have been considered for this case study: coarser, regular, finer and extra fine meshes, as shown in Fig. 13, where mesh elements are progressively reduced for correctly modeling the heat transfer mechanisms involved in the air gaps inside the tubes. The results of this analysis are shown in Fig. 14, where the maximum temperatures obtained for the conductor, shield, and jacked are represented as a function of the mesh size.

As can be seen, using a coarser mesh leads to high errors in the temperature estimation, while a regular mesh provides a good balance between results and simulation time. Finally, using a finer mesh or an extra fine mesh provides more accurate results, but with the main drawback of the computational cost. Consequently, in this study, a regular mesh is considered.

#### Size of the soil domain

**Fig. 15:**
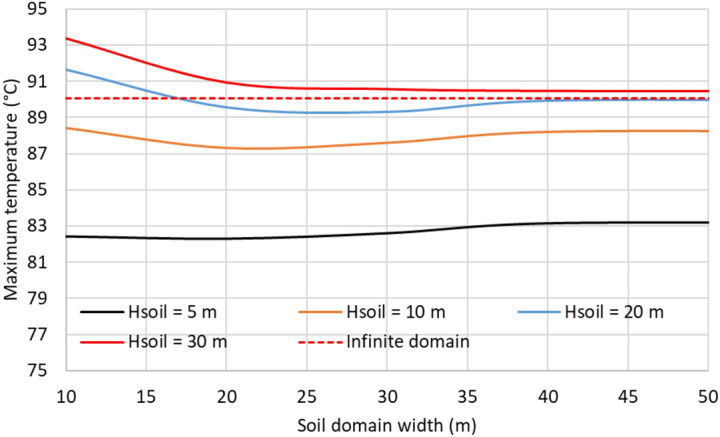
Influence of soil domain height and width on the maximum temperature of the conductors (phase current of 883 A).

**Fig. 16:**
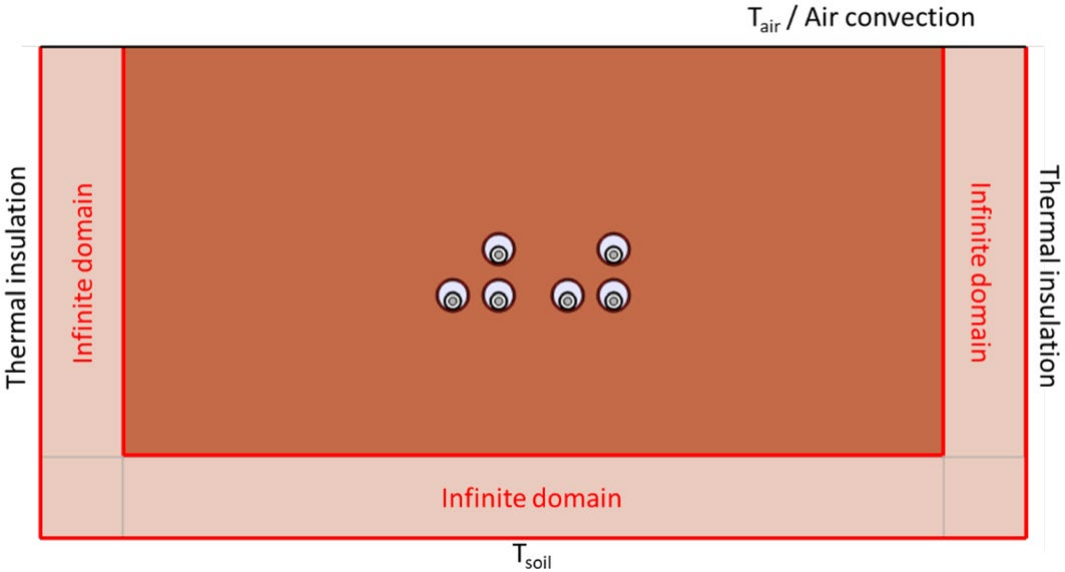
Use of the “Infinite domain element” feature in the soil domain.

The domain size employed for modeling the soil greatly affects the results so that an appropriate size may lead to incorrect results. In this sense, Wsoil and Hsoil have been modified to show this influence on the resulting maximum temperature of the conductors. Results are summarized in Fig. 15, where it can be easily concluded that results are more accurate as the soil dimensions increase, especially the Hsoil. However, this also concludes that Wsoil and Hsoil must be at least 40 m and 20 m, respectively. To avoid using such large dimensions, Comsol Multiphysics includes a feature called “Infinite element domain.” This feature can be applied to the soil’s most external layers (“Infinite domain” in Fig. 16), having the effect of stretching it to almost infinity^10^. Consequently, the model’s size is reduced, hence its computational cost. The result derived from this assumption is also included in Fig. 15 (red dashed line).

In the following, this feature is employed for the parametric analysis, where Wsoil and Hsoil are reduced to 6 m and 3 m, respectively.

#### Boundary conditions

**Fig. 17:**
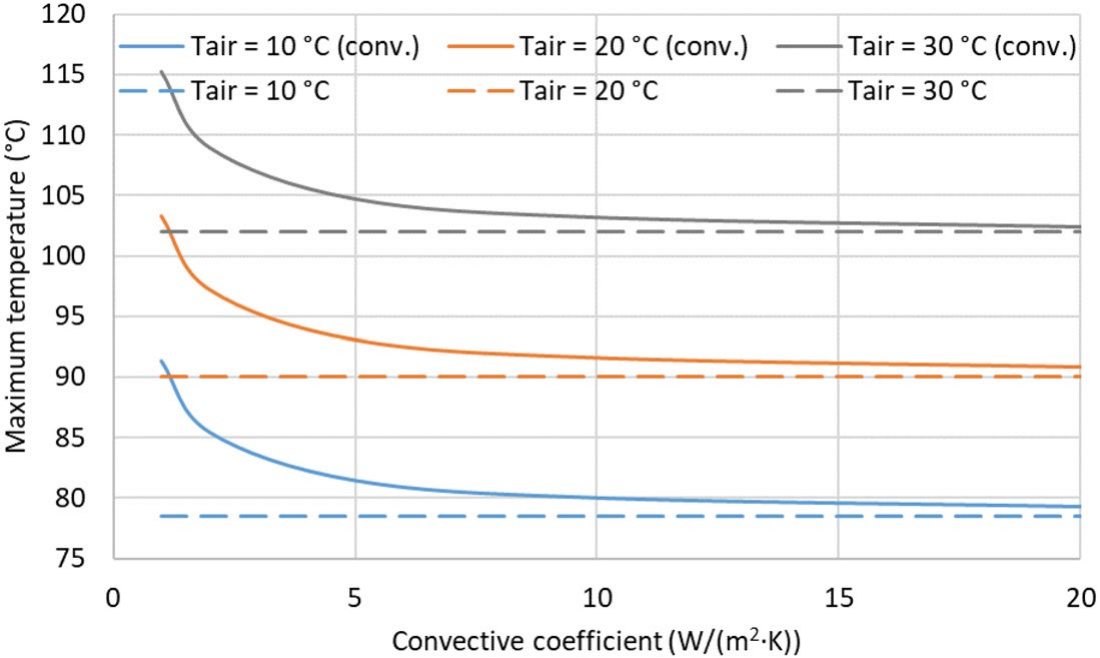
Influence of air temperature and convection coefficient in the maximum temperature of the conductors (phase current of 883 A).

Regarding the boundary conditions to be applied on the top and bottom boundaries of the system, it is commonly accepted to fix their temperatures at a certain value. Thus, on one hand, it is well-known that the soil temperature remains stable for a certain depth, so fixing the bottom boundary to Tsoil ensures this assumption, providing an alternative cold sink for the thermal problem. On the other hand, to fix the air-soil interface temperature to Tair entails with the Kennelly’s hypothesis considered in the IEC 60287 standard^2^. However, it is also possible to include the influence of the air convection mechanism on this boundary, defined by:

2 $$\begin{aligned} Q_{conv}=h \cdot (T-T_{air}) \end{aligned}$$

being T the temperature at the air-soil interface and h the convection coefficient in W/(m^2^ K). The impact of these boundary conditions, as well as their main parameters (h and Tair), are shown in Fig. 17, where the solid lines (denoted with “conv.”) are the cases where air convection is considered, using dashed lines for those where Tair is fixed at the air-soil interface.

As expected, the maximum temperature of the conductors increases with Tair. However, different behavior is observed depending on the boundary condition applied to the air-soi interface. Thus, as forced convection is more prominent (greater values for h), the resulting maximum temperature gets closer to that derived by fixing the temperature at the air-soil interface. Therefore, when forced convection is expected (h > 15 ${\text{W}}/({\text{m}}^2 \; {\text{K}})$.), there are no essential differences in applying any of these boundary conditions. Conversely, from this fig., it can also be concluded that air convection should be carefully considered when natural convection is expected in the actual location, especially for h < 5 ${\text{W}}/({\text{m}}^2 \; {\text{K}})$. Hence, for the parametric analysis developed next, Tair is fixed at the air-soil interface.

### Cable configuration

Once all aspects regarding the FEM model are properly set up, it is now possible to analyse the impact of different aspects of the cable configuration. In this sense, relevant aspects such as the phase arrangement, depth of burial, phases and circuits clearance, as well as the influence of the surrounding medium thermal properties.

#### Phases arrangement

**Fig. 18:**
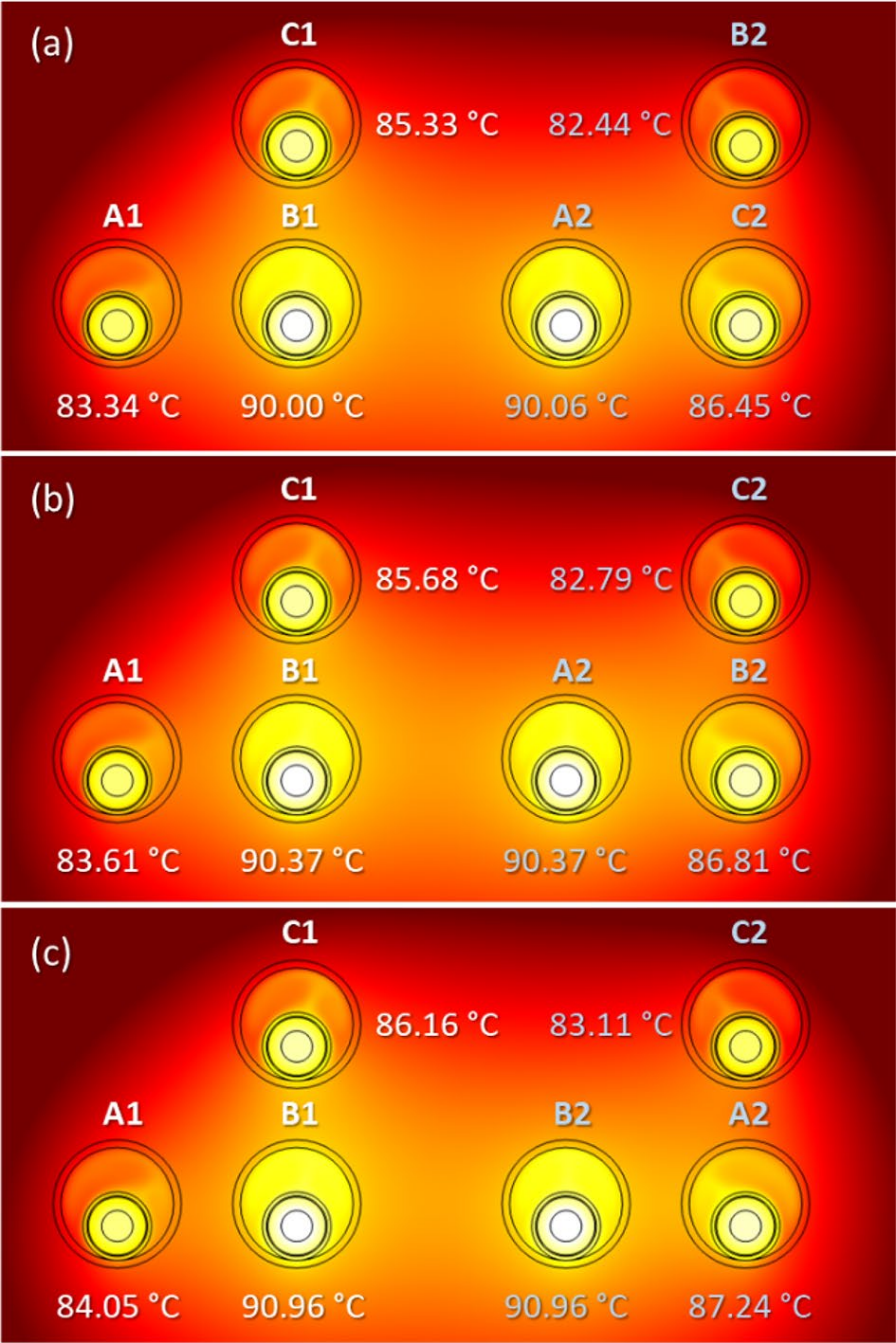
Maximum temperature in the conductors for different phase arrangements (phase current of 883 A).

The arrangement of the phases may also influence the maximum temperature achieved in the conductors. This is shown in Fig. 18 for three different phase arrangements, where differences about 1 °C are observed in the worst situation (cases shown in Fig. 18a and c), and therefore with almost no impact on the power line ampacity.

**Fig. 19:**
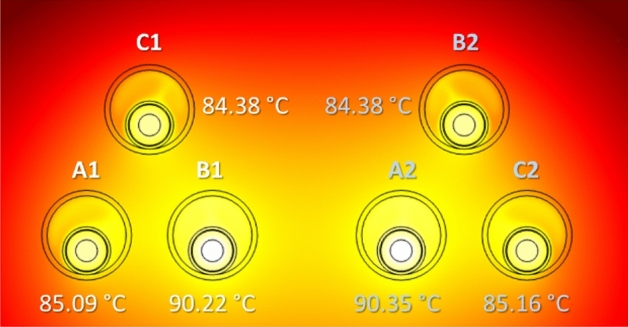
Maximum temperature in the conductors for two circuits in trefoil configuration (phase current of 883 A).

An alternative layout commonly employed in two-circuits underground power lines is that where each circuit is laid in trefoil configuration, as shown in Fig. 19. As can be seen, this configuration results in slightly higher temperature values (less than 0.5 °C in the hottest cable) when compared to the reference case (Fig. 18a), hence barely affecting the current rating that results in 880 A.

Consequently, given the phases and circuits clearances, the phases arrangement has no important effects on the power line ampacity.

#### Tubes in a concrete block

**Fig. 20:**
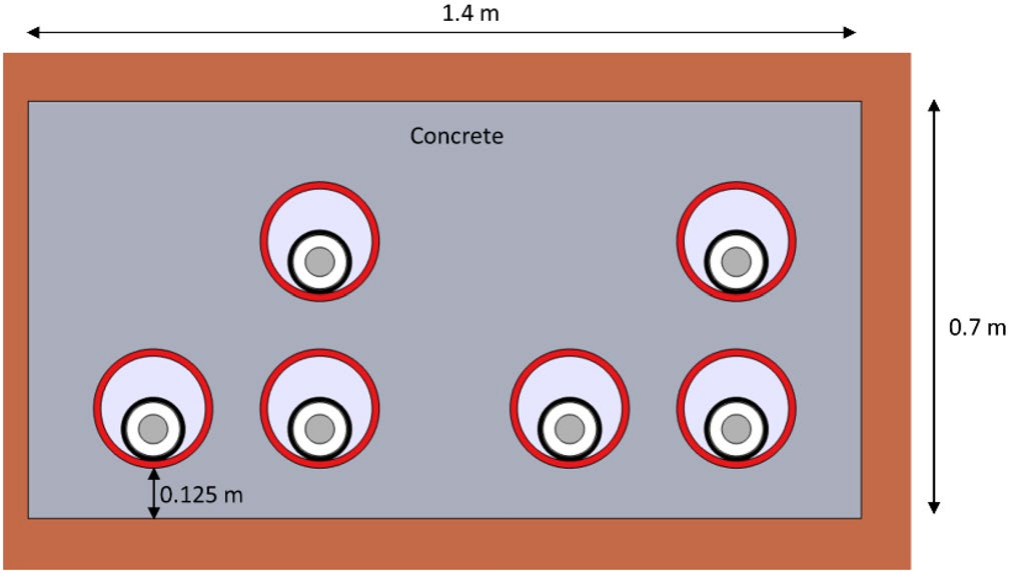
Dimensions of the concrete duct bank for the case study.

An important aspect that improves the thermal performance of underground power lines is the use of a concrete block when installing the tubes. To analyse its impact on the ampacity a 1.7 m × 0.7 m concrete block is employed in the reference case (Fig. 20), with a thermal conductivity of 1.177 ${\text{W}}/({\text{m}}^2 \; {\text{K}})$.

**Fig. 21:**
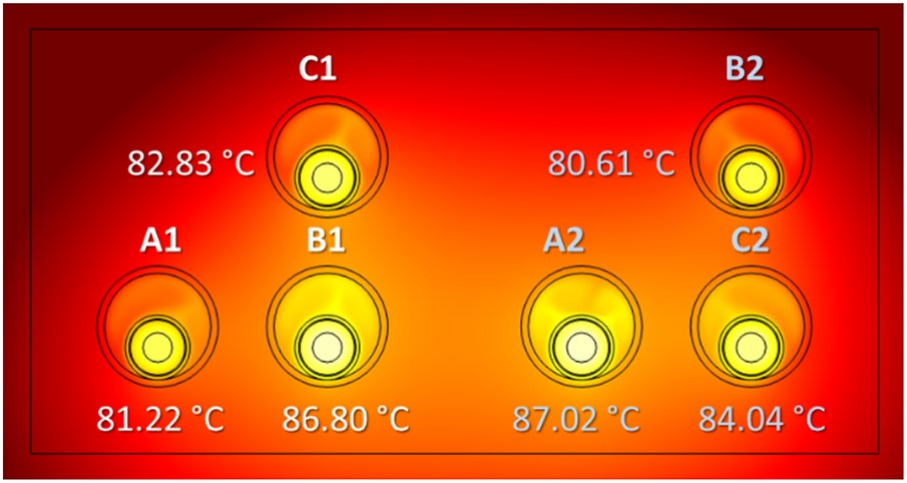
Maximum temperature of the conductors in a concrete duct bank (reference case study) (phase current of 883 A).

In this new configuration, the maximum temperature achieved in the conductors (Fig. 21) is lower than in the reference case (Fig. 18a), so the ampacity rises up to 903 A. This is due to the thermal conductivity of the concrete, that is higher than that of the surrounding soil, enhancing the heat dissipation of the system.

**Fig. 22:**
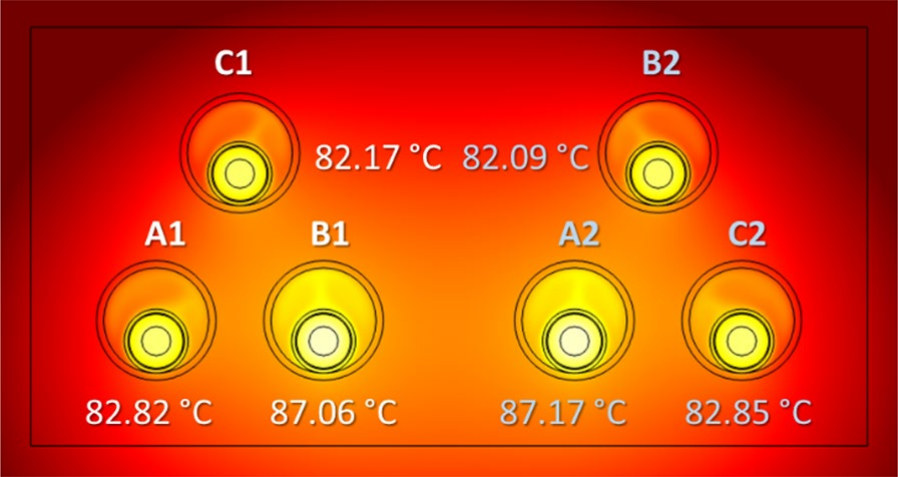
Maximum temperature of the conductors in a concrete duct bank (trefoil configuration) (phase current of 883 A).

This analysis is also applicable for the trefoil configuration considered in the previous section. In this case, lower values are also obtained for the maximum temperature (Fig. 22) in comparison with those obtained without the presence of the concrete block (Fig. 19), resulting in a line ampacity of 900 A.

The results for the different configurations considered in this analysis, either with or without concrete block, are summarized in Table 5, which included the resulting ampacity (Imax) for each situation.

**Table 5:** Summary of phase temperatures and ampacity for different phase arrangements.

Max. temperature	Ref. conf.	Trefoil	Ref. conf. (concr.)	Trefoil (concr.)
$$T_{A1} $$($$^\circ $$C)	83.34	85.09	81.22	82.81
$$T_{B1} $$($$^\circ $$C)	90	90.22	86.80	87.06
$$T_{C1} $$($$^\circ $$C)	85.33	84.38	82.83	82.17
$$T_{A2} $$($$^\circ $$C)	90.06	90.35	87.02	87.17
$$T_{B2} $$($$^\circ $$C)	82.44	84.38	80.61	82.09
$$T_{C2} $$($$^\circ $$C)	86.45	85.16	84.04	82.85
$I_{max}$(A)	883	880	903	900

#### Circuit’s and phase’s clearances

**Fig. 23:**
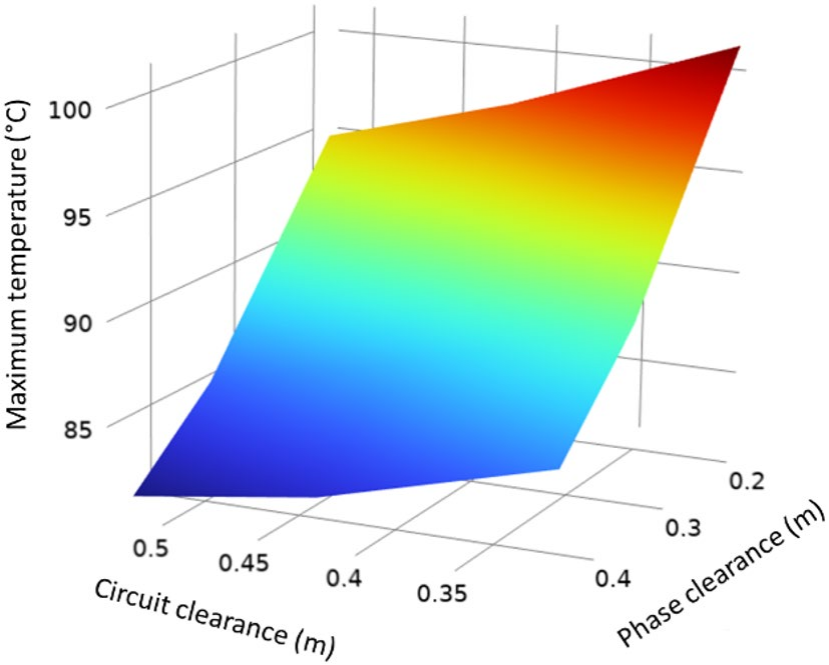
Influence of the clearance between phases and circuits in the conductor’s maximum temperature (phase current of 883 A).

The clearance between phases and circuits (s and dc in Fig. 23) is very important in thermal heating. The influence of both parameters on the maximum temperature achieved in the phase conductors is shown in Fig. 23. As expected, the temperature rises when reducing these clearances, making the influence of the phase’s clearance more prominent. Consequently, appropriate values must be found for both clearances within a reasonable range to reduce the impact of the mutual heating on the conductor’s temperature.

#### Burial conditions

**Fig. 24:**
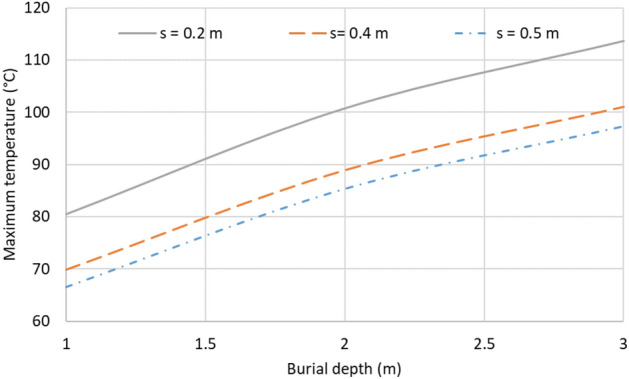
Influence on the conductor’s maximum temperature of the burial depth for different values of clearance between phases (phase current of 883 A).

Finally, burial conditions are a crucial point regarding the current rating of underground power lines. In this sense, two parameters are now analysed: the burial depth (db) and the thermal conductivity of the surrounding medium. Thus, Fig. 24 shows the impact of db on the maximum temperature achieved in the conductors for different clearance values between phases (s). As can be observed, a deeper installation leads to the hottest cables. However, this influence may be partially neutralized by choosing a suitable clearance between phases (in Fig. 24 the same maximum temperature is obtained for db = 1 m, s = 0.2 m, and db = 1.5 m, s = 0.4 m) together with the clearance between circuits, as observed in the previous section.

**Fig. 25:**
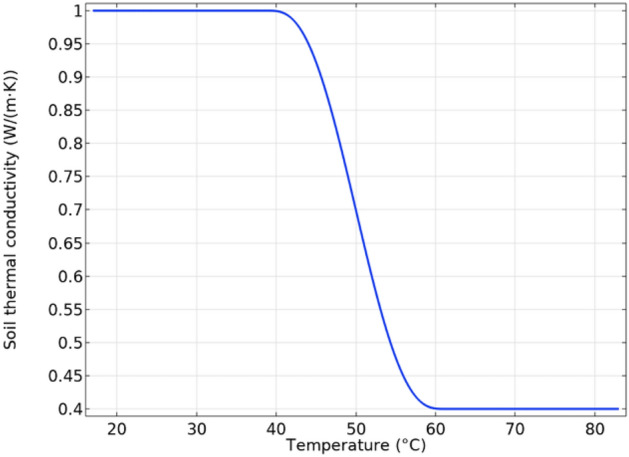
Surrounding medium thermal conductivity as a function of its temperature for considering the soil dryout.

Regarding the thermal conductivity of the surrounding medium, it is important to take into account its dependence on the moisture content since the heat flux caused by the power cables reduces the moisture content in the vicinity of the cables, resulting in a dried-out zone with worse thermal properties^11^. This is typically considered in the IEC 60287 standard^2^ through a certain value for the soil temperature (critical temperature) that delimits the change in the soil thermal conductivity. This can be modeled in COMSOL through a variable thermal conductivity as a function of its temperature^14^, as shown in Fig. 25, where the thermal conductivity ranges from 1 to 0.4 W/(m K) when considering a critical temperature of about 50 °C.

**Fig. 26:**
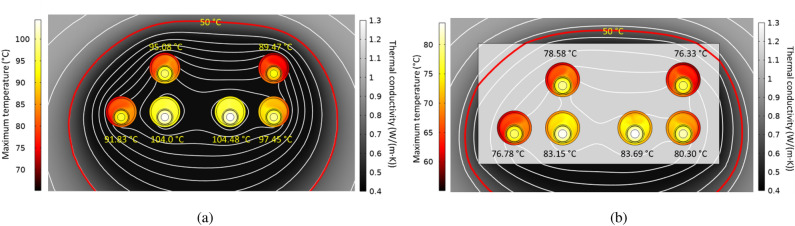
Maximum temperature in conductors, isotherms, and thermal conductivity in the surrounding medium for the reference case (phase current of 883 A).

The results obtained when considering this variable thermal conductivity for the soil are shown in Fig. 26. In this sense, Fig. 26a shows the temperature distribution in conductors and tubes, the isotherms, and the effective thermal conductivity at each location in the surrounding medium. The darkest area shows the dried-out zone around the conductors, limited by the 50 °C isotherm (red line in Fig. 26a). This worsens the heat dissipation mechanism, hence increasing the maximum temperature achieved in the conductors. In particular, temperatures are higher than those obtained for a constant thermal conductivity of 0.833 W/(m K) (Fig. 18a), even though the variable thermal conductivity of Fig. 25 can reach up to 1 W/(m K).

Conversely, when a concrete block is present (Fig. 26b), the moisture migration phenomenon only occurs out of the concrete block, limited to a minor extension than in the previous case; this way, conductors are surrounded by a favorable thermal medium that facilitates the heat evacuation mechanism, this situation, together with the fact that out of the dried-out zone the thermal conductivity is 1 W/(m K), leads to temperatures below those derived using a constant thermal conductivity (Fig. 21).

## Conclusion

In conclusion, the numerical and experimental analysis of the thermal behavior of high-voltage power cables in unfilled ducts is an essential area of research that has significant implications for the sustainability of electrical energy distribution systems. The results of our study indicate that the temperature distribution within the cable is strongly influenced by the position of the duct, its environment, and the thermal properties of the materials used in the construction of the cable. The numerical simulations performed in this study provide a valuable tool for predicting the temperature distribution within the cable. They can be used to optimize the design of high-voltage power cables for use in unfilled ducts. In Table 6, the values of the percentage errors between simulation and experimental data are reported. The reported values highlight that the simulations faithfully reproduce the actual values. The experimental measurements obtained from our study validate the accuracy of the numerical simulations and provide a basis for further investigation into the thermal behavior of high-voltage power cables. Overall, the findings of this study have important implications for the design and operation of high-voltage power cables in unfilled ducts. By improving our understanding of the thermal behavior of these cables, we can develop a more efficient and sustainable electrical energy distribution infrastructure that can meet the growing demand for electricity while minimizing environmental impact. Future research in this area should focus on developing more accurate and efficient numerical models for predicting the temperature distribution within high-voltage power cables and exploring new materials and designs that can improve their thermal performance.

**Table 6:** Values of percentage errors on the different measurement points for different current value.

Case	1000 A centered error [%]	1000 A resting error [%]	1250 A centered error [%]	1250 A resting error [%]
Conductor	0.4	0.8	0.4	4
Shield	0.3	2.7	0.5	3
Insulation	3.6	2.9	5	6.6
Pipe outer wall	7.3	5	4	4.1
Pipe internal wall	1.2	4	0	7

## Data Availability

All data generated or analysed during this study are included in this published article.
